# Biomarker Extraction Based on Subspace Learning for the Prediction of Mild Cognitive Impairment Conversion

**DOI:** 10.1155/2021/5531940

**Published:** 2021-09-02

**Authors:** Ying Li, Yixian Fang, Jiankun Wang, Huaxiang Zhang, Bin Hu

**Affiliations:** ^1^Key Laboratory of TCM Data Cloud Service in Universities of Shandong, Shandong Management University, Jinan 250357, China; ^2^School of Information Science and Engineering, Shandong Normal University, Jinan 250358, China; ^3^School of Mathematics and Statistics, Qilu University of Technology, Jinan 250353, China; ^4^Shandong Big Data Center, Jinan 250011, China; ^5^Key Laboratory of Wearable Computing of Gansu Province, Lanzhou University, Lanzhou 730000, China

## Abstract

Accurate recognition of progressive mild cognitive impairment (MCI) is helpful to reduce the risk of developing Alzheimer's disease (AD). However, it is still challenging to extract effective biomarkers from multivariate brain structural magnetic resonance imaging (MRI) features to accurately differentiate the progressive MCI from stable MCI. We develop novel biomarkers by combining subspace learning methods with the information of AD as well as normal control (NC) subjects for the prediction of MCI conversion using multivariate structural MRI data. Specifically, we first learn two projection matrices to map multivariate structural MRI data into a common label subspace for AD and NC subjects, where the original data structure and the one-to-one correspondence between multiple variables are kept as much as possible. Afterwards, the multivariate structural MRI features of MCI subjects are mapped into a common subspace according to the projection matrices. We then perform the self-weighted operation and weighted fusion on the features in common subspace to extract the novel biomarkers for MCI subjects. The proposed biomarkers are tested on Alzheimer's Disease Neuroimaging Initiative (ADNI) dataset. Experimental results indicate that our proposed biomarkers outperform the competing biomarkers on the discrimination between progressive MCI and stable MCI. And the improvement from the proposed biomarkers is not limited to a particular classifier. Moreover, the results also confirm that the information of AD and NC subjects is conducive to predicting conversion from MCI to AD. In conclusion, we find a good representation of brain features from high-dimensional MRI data, which exhibits promising performance for predicting conversion from MCI to AD.

## 1. Introduction

Alzheimer's disease (AD) characterized by memory loss and cognitive decline is the most prevalent neurodegenerative disease [1, 2]. Mild cognitive impairment (MCI) is regarded as the prodromal stage of AD with possibility to develop AD. Individuals with MCI can carry out daily activities, but their thinking abilities have mild and measurable changes [3]. On average, 32 percent of individuals with MCI will convert to AD within 5 years [4]. Therefore, it is critical to identify MCI as early as possible, so that we can delay the progress of AD by the well-targeted treatment. The development of neuroimaging techniques provides powerful tools for early prediction of AD. Structural magnetic resonance imaging (MRI) with high spatial resolution, high availability, noninvasive nature, and moderate costs is an extensively used neuroimaging modality. Numerous structural MRI-based biomarkers have been extracted for the AD detection at different stages [5–13]. For instance, in [6], spatial frequency components of cortical thickness were used for individual AD identification based on incremental learning. In [13], an individual network was constructed using six types of morphological features to improve the accuracy of AD and MCI diagnoses. However, since the pathological variations are subtle at the MCI stage, it is still challenging to develop more advanced biomarkers to accurately predict the conversion from MCI to AD.

According to whether the MCI subjects will convert to AD or not within a given time period (i.e., 3 years), they are separated into two categories: progressive MCI (pMCI) and stable MCI (sMCI). Previous studies [14, 15] have shown that the subjects with pMCI are similar to AD while subjects with sMCI are more like normal control (NC). As a result, the classification between AD and NC is a simple version of that between pMCI and sMCI. Due to the high heterogeneity of MCI population, it is effective to take advantage of AD and NC information in MCI conversion prediction, such as feature selection and classifier training. Studies [14–22] also have demonstrated that the information of AD and NC subjects is helpful in distinguishing pMCI subjects from sMCI subjects. In [16, 17], the data of AD and NC subjects was used to build classifier for the discrimination between pMCI and sMCI subjects. In [18–20], the AD and NC subjects were regarded as labeled samples while MCI subjects were taken as unlabeled samples, and a semisupervised learning approach was applied to dividing MCI subjects into normal-like and AD-like categories. In [14], to distinguish pMCI from sMCI, a semisupervised low-density separation (LDS) method was used to integrate AD and NC information. In [21], a novel domain transfer learning method drawing support from AD and NC subjects was used for MCI conversion prediction. Besides, some studies extracted novel biomarkers for MCI conversion prediction by information propagation from AD and NC subjects to MCI subjects. For instance, in [22], the information was propagated from AD and NC subjects to MCI subjects by a weighting function, and the average grading value was computed for MCI classification. In [15], the disease labels of AD and NC subjects were propagated to MCI subjects using elastic net technique, and a global grading biomarker was developed.

Owing to the high dimensionality of MRI features, it is difficult to find a good representation of brain features to reveal their subtle pathological variations for MCI conversion prediction [23]. The subspace learning method as a dimension reduction approach has become a hot topic in many fields [24–30]. In the field of AD diagnosis, several subspace learning methods, such as canonical correlation analysis (CCA) [31, 32], independent component analysis (ICA) [33, 34], partial least squares (PLS) [35, 36], locality preserving projection (LPP) [37, 38], linear discriminant analysis (LDA) [38, 39], and locally linear embedding (LLE) [23, 40], have demonstrated promising performance. For instance, in [23], multivariate MRI data were transformed into a locally linear space by LLE algorithm, and the embedded features were used to predict the conversion from MCI to AD. In [34], the risk factors associated with MCI conversion were investigated by combining ICA with the multivariate Cox proportional hazards regression model. In [38], a sparse least square regression framework with LDA and LPP was proposed for feature selection in AD diagnosis. The experimental results verified that subspace learning methods outperformed feature selection methods. Although many subspace learning methods have been applied to the early detection of AD, it is still a challenging problem to map MRI data into a low-dimensional subspace and find representative brain features for detecting the differences between pMCI and sMCI. In addition, it is interesting to investigate how the AD and NC data can provide auxiliary information in this procedure and enhance the performance of MCI classification.

In this work, we propose a method to extract biomarkers of MCI subjects based on subspace learning for predicting conversion from MCI to AD. Specifically, we first learn two projection matrices to map multivariate MRI data of regional cortical thickness (CT) and cortical volume (CV) into a common label subspace with lower dimensions for AD and NC subjects, where the correlation of multiple variables and the original data structure are kept as much as possible. We then use the projection matrices to map the CT and CV data of the MCI subjects into the common subspace to obtain the CT- and CV-based features for MCI subjects accordingly. After that, we perform self-weighted operation and weighted fusion on the CT- and CV-based features in common subspace and extract the novel biomarkers for MCI subjects.

## 2. Materials and Method

### 2.1. Image Data and Preprocessing

Data used in this work are acquired from Alzheimer's Disease Neuroimaging Initiative (ADNI) database (http://adni.loni.usc.edu/). We use baseline MRI scans (1.5 T, 1.25 mm × 1.25 mm in-plane spatial resolution, 1.2 mm thick slices) of 528 subjects, which include 142 AD subjects, 165 NC subjects, and 221 MCI subjects. Moreover, the 221 MCI subjects contain 126 pMCI and 95 sMCI subjects. The characteristics of the participants are shown in Table 1.

The image preprocessing involves the following steps: motion correction, nonbrain tissue removal, coordinate transformation, gray matter (GM) segmentation, and reconstruction of GM/white matter boundaries [41–43]. We conducted all preprocessing steps by FreeSurfer v5.3.0 (http://surfer.nmr.mgh.harvard.edu). The reconstruction and segmentation errors are visually checked using FreeView software and manually corrected. After that, surface inflation and registration are performed, followed by cortical thickness and volume measurement calculation [44]. Finally, the images were smoothed by a 30 mm full width at half maximum Gaussian kernel [45]. The images are segmented into 90 regions in the light of the automated anatomical labeling atlas [46], and then, 12 subcortical regions are removed owing to the lack of the thickness features. The average cortical thickness and cortical volume of each region are calculated and used as features.

### 2.2. Method

**Figure 1:**
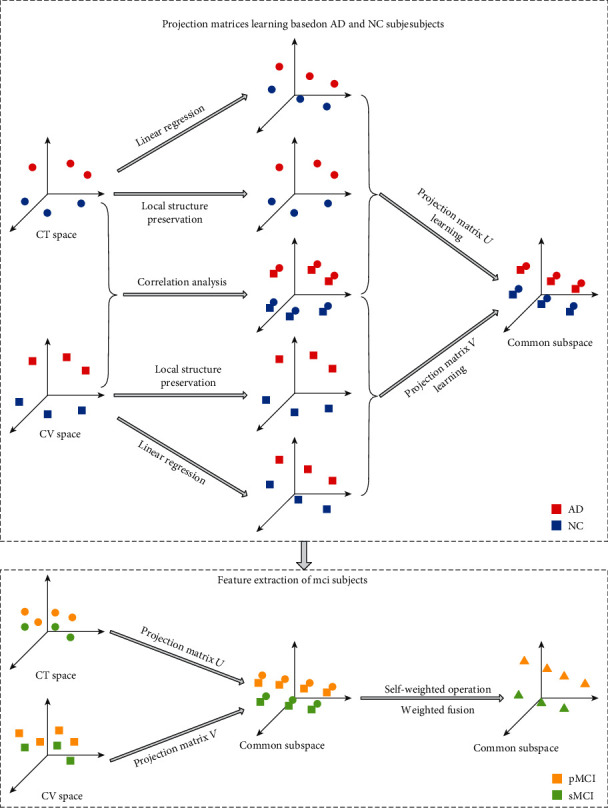
Schematic representation of the proposed method.

Schematic representation of our proposed method is provided in Figure 1. The method includes three steps: (1) Taking AD and NC subjects as auxiliary data, we learn two projection matrices. (2) The MCI subjects are mapped into subspace according to the projection matrices. (3) Self-weighted operation and weighted fusion are performed on the features in the subspace, and the biomarkers are extracted.

#### 2.2.1. Learning Projection Matrices Using Auxiliary Data

In this subsection, with AD and NC subjects as auxiliary data, we learn two projection matrices to map multivariate structural MRI data of regional cortical thickness and volume into a common label subspace, where the original data structure and the one-to-one correspondence between multiple variables are kept as much as possible. Let*X*_CT_ = [*x*_1_^CT^, *x*_2_^CT^, ⋯, *x*_*n*_^CT^] ∈ ℝ^*d*×*n*^ and *X*_CV_ = [*x*_1_^CV^, *x*_2_^CV^, ⋯, *x*_*n*_^CV^] ∈ ℝ^*d*×*n*^ denote the cortical thickness and cortical volume feature matrices, respectively, where *n* is the number of AD and NC subjects, and *d* is the number of feature dimensions. Let *Y* ∈ ℝ^*n*×*c*^ represent a class indicator matrix with 0-1 encoding, where *c* is the number of classes. To learn the two projection matrices *U*^*d*×*c*^ and *V*^*d*×*c*^, the objective function is defined as follows:

(1) $$\begin{array}{c} \underset{U,V}{\min}Q\left(U,V\right)=\lambda l\left(U,V\right)+\left(1-\lambda \right)f\left(U,V\right)+\alpha g\left(U,V\right)+\beta r\left(U,V\right). \end{array}$$

The first term *l*(*U*, *V*) is the linear regression from the feature space to the label space, and it guarantees that samples are close to their labels after projection. *l*(*U*, *V*) is expressed as follows:

(2) $$\begin{array}{c} l\left(U,V\right)={\left\|Y-X_{\text{CT}}^{T}U\right\|}_{F}^{2}+{\left\|Y-X_{\text{CV}}^{T}V\right\|}_{F}^{2}. \end{array}$$

The second term maintains the correlation between the CT features and CV features of the same image. It is well known that different morphological features of the same image reflect the same label information from different views. They should be close to each other after projection. Therefore, *f*(*U*, *V*) is defined as follows:

(3) $$\begin{array}{c} f\left(U,V\right)={\left\|X_{\text{CT}}^{T}U-X_{\text{CV}}^{T}V\right\|}_{F}^{2}. \end{array}$$

The third term *g*(*U*, *V*) is the graph regularization term, which is used to better exploit the local structural information of the data. We aim to preserve the neighborhood relationship between samples of single morphological feature. Here, we first introduce the graph regularization term for cortical thickness feature *X*_CT_. We define an undirected and symmetric graph *G*_CT_ = (*V*_CT_, *W*_CT_), where *V*_CT_ is a collection of samples in *X*_CT_ and *W*_CT_ represents the relations between samples. Each element *w*_*ij*_^CT^ in *W*_CT_ is defined as follows:

(4) $$\begin{array}{c} w_{ij}^{\text{CT}}=\left\{\begin{array}{cc} \exp\left(-\frac{{\left(x_{i}^{\text{CT}}-x_{j}^{\text{CT}}\right)}^{2}}{2\sigma^{2}}\right), & \text{if }x_{i}^{\text{CT}}\in N_{k}\left(x_{j}^{\text{CT}}\right),i\neq j,\\ 0, & \text{otherwise}, \end{array}\right. \end{array}$$

where *N*_*k*_(*x*_*j*_^CT^) denotes the *k*-nearest neighbors of *x*_*j*_^CT^. Let *a*_*i*_ denote the *i*-th column of *U*^*T*^*X*_CT_; then, the graph regularization term for cortical thickness data is formulated as follows:

(5) $$\begin{array}{c} g_{\text{CT}}\left(U\right)=\frac{1}{2}\overset{n}{\underset{i,j=1}{\sum }}{\left\|a_{i}-a_{j}\right\|}_{2}^{2}w_{ij}^{\text{CT}}=tr\left(U^{T}X_{\text{CT}}L_{\text{CT}}X_{\text{CT}}^{T}U\right), \end{array}$$

where *L*_CT_ = *D*_CT_ − *W*_CT_ is the graph Laplacian matrix and *D*_CT_ ∈ ℝ^*n*×*n*^ is a diagonal matrix with its diagonal elements *D*_*ii*_^CT^ = ∑_*j*_*w*_*ij*_^CT^.

Similarly, for the cortical volume data *X*_CV_, let *b*_*i*_ denote the *i*-th column of *V*^*T*^*X*_CV_. The graph regularization term of volume data is formulated as follows:

(6) $$\begin{array}{c} g_{\text{CV}}\left(V\right)=\frac{1}{2}\overset{n}{\underset{i,j=1}{\sum }}{\left\|b_{i}-b_{j}\right\|}_{2}^{2}w_{ij}^{\text{CV}}=tr\left(V^{T}X_{\text{CV}}L_{\text{CV}}X_{\text{CV}}^{T}V\right), \end{array}$$

where *w*_*ij*_^CV^ and *L*_CV_ are defined as before. The final representation of the graph regularization term is then given by the following:

(7) $$\begin{array}{c} g\left(U,V\right)=g_{\text{CT}}\left(U\right)+g_{\text{CV}}\left(V\right)=tr\left(U^{T}X_{\text{CT}}L_{\text{CT}}X_{\text{CT}}^{T}U\right)+tr\left(V^{T}X_{\text{CV}}L_{\text{CV}}X_{\text{CV}}^{T}V\right). \end{array}$$

The last term *r*(*U*, *V*) controls the scale of projection matrices and avoids overfitting:

(8) $$\begin{array}{c} r\left(U,V\right)={\left\|U\right\|}_{F}^{2}+{\left\|V\right\|}_{F}^{2}. \end{array}$$

Besides, *λ*, *α*, and *β* are the three balancing parameters. Based on Equations (2), (3), (7), and (8), we can obtain the final objective function as follows:

(9) $$\begin{array}{c} \underset{U,V}{\min}Q\left(U,V\right)=\lambda \left({\left\|Y-X_{\text{CT}}^{T}U\right\|}_{F}^{2}+{\left\|Y-X_{\text{CV}}^{T}V\right\|}_{F}^{2}\right)+\left(1-\lambda \right)\left({\left\|X_{\text{CT}}^{T}U-X_{\text{CV}}^{T}V\right\|}_{F}^{2}\right)+\alpha \left[tr\left(U^{T}X_{\text{CT}}L_{\text{CT}}X_{\text{CT}}^{T}U\right)+tr\left(V^{T}X_{\text{CV}}L_{\text{CV}}X_{\text{CV}}^{T}V\right)\right]+\beta \left({\left\|U\right\|}_{F}^{2}+{\left\|V\right\|}_{F}^{2}\right). \end{array}$$

#### 2.2.2. Optimization Algorithm

Both *U* and *V* are initialized as zero matrices. We then iteratively update each variable by fixing another variable. By setting the partial derivative of *Q*(*U*, *V*) with respect to *U* and setting it to zero, we have the following:

(10) $$\begin{array}{c} \frac{\partial \left(U,V\right)}{\partial U}=2\lambda \left(X_{\text{CT}}X_{\text{CT}}^{T}U-X_{\text{CT}}Y\right)+2\left(1-\lambda \right)\left(X_{\text{CT}}X_{\text{CT}}^{T}U-X_{\text{CT}}X_{\text{CV}}^{T}V\right)+2\alpha X_{\text{CT}}L_{\text{CT}}X_{\text{CT}}^{T}U+2\beta U=0. \end{array}$$

We can get the following:

(11) $$\begin{array}{c} U={\left(X_{\text{CT}}X_{\text{CT}}^{T}+\alpha X_{\text{CT}}L_{\text{CT}}X_{\text{CT}}^{T}+\beta I\right)}^{-1}\left[\lambda X_{\text{CT}}Y+\left(1-\lambda \right)X_{\text{CT}}X_{\text{CV}}^{T}V\right]. \end{array}$$

Similarly, by fixing *U* and updating *V*, we can obtain the following:

(12) $$\begin{array}{c} V={\left(X_{\text{CV}}X_{\text{CV}}^{T}+\alpha X_{\text{CV}}L_{\text{CV}}X_{\text{CV}}^{T}+\beta I\right)}^{-1}\left[\lambda X_{\text{CV}}Y+\left(1-\lambda \right)X_{\text{CV}}X_{\text{CT}}^{T}U\right]. \end{array}$$

The procedure of projection matrices learning with auxiliary data is described in Algorithm 1.

#### 2.2.3. Feature Extraction of MCI Subjects

Let *Z*_CT_ = [*z*_1_^CT^, *z*_2_^CT^, ⋯, *z*_*m*_^CT^] ∈ ℝ^*d*×*m*^ and *Z*_CV_ = [*z*_1_^CV^, *z*_2_^CV^, ⋯, *z*_*m*_^CV^] ∈ ℝ^*d*×*m*^ denote the cortical thickness and cortical volume feature matrices of the *m* images of MCI subjects, respectively. The feature representations of MCI subjects in subspace are denoted by Fea_CT_ ∈ ℝ^*m*×*c*^ and Fea_CV_ ∈ ℝ^*m*×*c*^, which are computed as follows:

(13) $$\begin{array}{c} {\text{Fea}}_{\text{CT}}=Z_{\text{CT}}^{T}\times U, \end{array}$$

(14) $$\begin{array}{c} {\text{Fea}}_{\text{CV}}=Z_{\text{CV}}^{T}\times V. \end{array}$$

To make the projected features of pMCI and sMCI subjects are more discriminative, as well as balance the effectiveness of features from thickness and volume data, we perform self-weighted operation and weighted fusion on the features in subspace to obtain the final features. Finally, the biomarkers for MCI subjects are defined as follows:

(15) $$\begin{array}{c} \text{Fea}=\eta *\left(\left|{\text{Fea}}_{\text{CT}}\right|*{\text{Fea}}_{\text{CT}}\right)+\left(1-\eta \right)*\left(\left|{\text{Fea}}_{\text{CV}}\right|*{\text{Fea}}_{\text{CV}}\right), \end{array}$$

where *η* is the weight parameter. |Fea_CT_| represents the absolute values of all elements in matrix Fea_CT_.

## 3. Experiments and Results

We first evaluated the performance of the proposed biomarkers by carrying out pairwise classifications with three classifiers, i.e., decision tree classifier, support vector machine (SVM) with RBF kernel, and SVM with linear kernel. To verify the efficacy of the feature reduction, the proposed method was also compared with four commonly used feature reduction methods. Second, we compared the performance of the proposed biomarkers with that of state-of-the-art methods. Third, the effectiveness of learning projection matrices using AD and NC information was validated. Finally, the discrimination ability of the proposed biomarkers was illustrated. To make fair comparisons, we repeated 10-fold cross-validation 20 times to report the average results for each method. The10-fold cross-validation strategy partitioned all samples into 10 subsets, left one subset for testing and other subsets for training until each of the 10 subsets was tested. Four measures including accuracy (ACC), sensitivity (SEN), specificity (SPE), and area under the receiver operating characteristic curve (AUC) were used to comprehensively evaluate the performance for all methods. Moreover, to assess whether the differences between the two competing methods were statistically significant, paired *t*-tests at 95% significance level were performed on the classification accuracies of the 20 runs.

We conducted all the experiments under MATLAB R2016b. Specifically, the decision tree classifier was implemented based on the MATLAB build-in functions. SVM with RBF kernel and linear kernel were adopted from the LIBSVM toolbox [47] and LIBLINEAR toolbox [48], respectively. For the three balancing parameters in Equation (9), *λ* was tested in the range of {0.1, 0.2, ⋯, 0.9}, while the parameter *α* was tested at the logarithmic scale of 10^*i*^ with *i* = {−3, −2, ⋯, 1}, and the parameter *β* was also determined at the logarithmic scale of 10^*j*^ with *j* = {−1, 0, 1}. The value of nearest neighbors *k* was tested from the set of {3, 5, 7, 9, 11, 13, 15}. Besides, the parameter *η* in Equation (15) was determined in a specific range (*η* ∈ {*q* × 10^−2^, *q* × 10^−1^}, where *q* ∈ {1, 2, ⋯, 9}). Note that we also conducted the parameter optimization for each method in comparison to reach their best performance.

### 3.1. Evaluation of Classification Performance

In this subsection, we first compared the classification performance of the proposed biomarkers with that of global grading biomarker in [15], based on three different classifiers, i.e., decision tree classifier, SVM with RBF kernel, and SVM with linear kernel. In [15], elastic net was used to propagate the information of AD and NC subjects to the target MCI subject, and a global grading biomarker was extracted for each MCI subject. We used the same method as proposed in [15] but calculated the grading biomarkers based on regionwise features. The sparse coding process of elastic net [49] was implemented via SPAMS toolbox [50].  Table 2 demonstrates the group classification results of the proposed biomarkers and the global grading biomarker developed in [15], separately for the three classifiers. The classification performances of our proposed biomarkers were significantly better (*p* < 0.05) than that of global grading biomarker in [15] under decision tree classifier and SVM with linear kernel. There was no significant difference in the classification performance between the two competitive biomarkers using SVM with RBF kernel, although the classification accuracy, sensitivity, specificity, and AUC of the proposed biomarkers were slightly higher. In conclusion, the proposed biomarkers were superior to or at least as good as the global grading biomarker in [15] under different classifiers. The proposed biomarker achieved highest accuracy of 69.37% when using SVM classifier with linear kernel.

**Figure 2:**
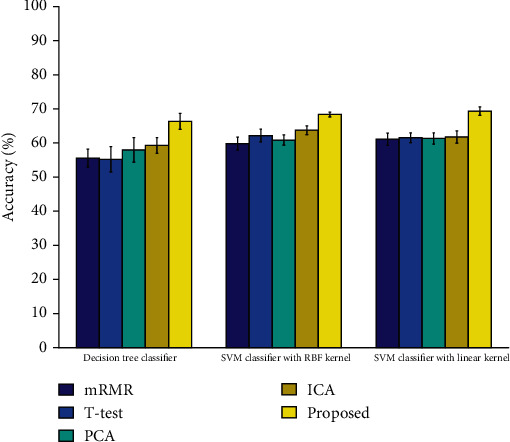
Comparison of different feature reduction methods.

As mentioned above, the proposed method could reduce the feature dimensions and extract meaningful biomarkers. To verify its performance on dimensionality reduction, we further compared the proposed method with four commonly used feature reduction methods, i.e., minimum redundancy and maximum relevance (mRMR) [51], *t*-test, principal component analysis (PCA) [52], and ICA. The mRMR method selects features according to the minimum redundancy and maximum relevance criterion based on mutual information. *t*-test is one of the statistical hypothesis testing techniques, which has been successfully used for supervised feature selection in neuroimaging studies [53]. Both PCA and ICA are subspace learning methods. PCA captures most of the variance in the data by linearly transforming correlated features into a smaller number of uncorrelated features. ICA separates data into a set of independent and relevant features. We compared above feature reduction methods with the three aforementioned classifiers. The best number of features for each competing method was found by grid search optimization. As can be seen from Figure 2, the proposed method outperformed other feature reduction methods with all three classifiers. The proposed method improved the classification accuracy on average by 9.21%, 8.38%, 7.97%, and 6.43% compared to mRMR, *t*-test, PCA, and ICA, respectively.

### 3.2. Comparison with State-of-the-Art Methods

In this subsection, we compared the best classification performance of the proposed biomarkers with that of the feature extraction methods presented in [13, 15] on the same dataset. In [13], the MFN features were extracted, and then, the two-step feature selection mRMR and SVM-based recursive feature elimination (SVM-RFE) [54] were employed to find the optimal MFN feature subset. Finally, the SVM classifier with RBF kernel was used for pMCI and sMCI classification. In [15], grading biomarkers were calculated using elastic net technique, and then, the SVM classifier with linear kernel was used for classification. In order to show the validity of our feature extraction strategy, the original morphological features were also added for comparison using the same feature selection strategy and classifier as literature [13].

Table 3 summarizes the classification results of all competing methods. It is notable from Table 3 that all feature extraction methods outperformed the method of exploiting original morphological features in terms of ACC, SPE, and AUC, which implies that the extraction of effective features can improve classification performance. In virtue of subspace learning, our proposed method achieved the highest classification accuracy and sensitivity among all competing methods. Specifically, compared with the methods proposed in [13, 15], our method improved the classification accuracy by 3.76% and 1.94% and improved the sensitivity by 4.76% and 5.35%, respectively. Therefore, it is reasonable to integrate subspace learning into the feature extraction, which can enhance the classification power of the features.

The best parameter combination found by experiments was *λ* = 0.1, *α* = 0.1, *β* = 10, and *η* = 0.03. The numbers of nearest neighbors for cortical thickness and volume data were 11 and 3, respectively. For the classification of pMCI and sMCI, the class indicator *c* = 2.

### 3.3. Effectiveness of Learning Projection Matrices Using AD and NC Information

In this subsection, we examined the effectiveness of learning projection matrices using AD and NC data. For comparison, we learned projection matrices by virtue of pMCI and sMCI data. The same procedure of MCI feature extraction as Section 2.2 was conducted. Three different classifiers, i.e., decision tree classifier, SVM with RBF kernel, and SVM with linear kernel, were used for test in turn. We also conducted 10-fold cross-validation for 20 times to obtain the average results. To be specific, we randomly divided the MCI dataset into 10 subsets and then iteratively left one subset for testing and the remaining 9 subsets for training until each of the 10 subsets was validated. The two projection matrices were learned from the training subsets, and then, all the data of training subsets and testing subsets were projected from original space into the subspace by the two projection matrices. At last, the biomarkers were computed according to Equation (15). All the parameters of the competing methods were optimized in the same range as our proposed method.

Table 4 demonstrates the classification results of learning projection matrices using different data. Compared with pMCI and sMCI data, the projection matrices learned with AD and NC data obtained better classification performance no matter which classifier was used. In particular, compared to learning projection matrices using pMCI and sMCI data, the proposed method obtained significant improvements on the classification accuracy and sensitivity by 4.59% and 7.8% when using SVM classifier with linear kernel, respectively. These results confirmed the efficacy of adopting AD and NC data in the subspace learning in our method. Meanwhile, this also validated that the inclusion of AD and NC information is beneficial for the classification between pMCI and sMCI [14, 15, 17, 19, 21, 22, 55].

### 3.4. Visualization

**Figure 3:**
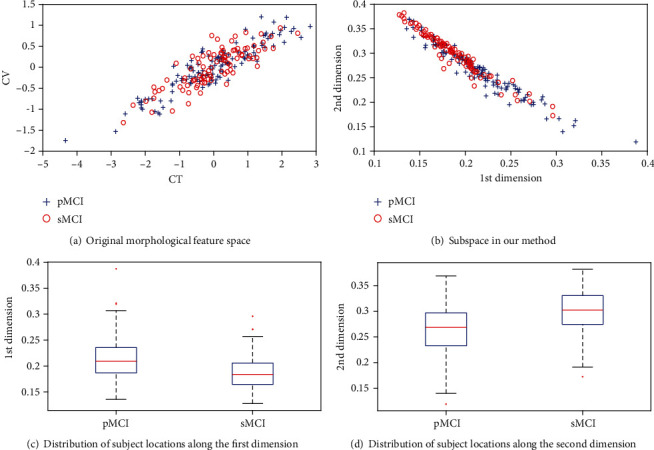
Visualization of all MCI samples in original feature space and subspace. (a) The distributions of pMCI and sMCI samples in original morphological feature space. (b) The distributions of pMCI and sMCI samples in subspace. (c, d) The distribution of subject locations by group along the first and second dimensions, respectively.

In this subsection, we illustrated the distributions of MCI samples in original morphological feature space and the projected subspace, respectively, to visually exhibit the distinguishing ability of different features. For the original morphological features, the PCA was applied to converting the original thickness and volume features to a number of uncorrelated features, respectively. Here, we employed the first principal component with the largest amount of variance for each type of morphological features and displayed the sample distribution in the two-dimensional space. In the original feature space (Figure 3(a)), it is clear to see that the distributions of pMCI and sMCI samples overlapped severely and samples in each class were scattered. Thus, the classification performance of the original features was very limited. In contrast, interclass distance of the pMCI and sMCI samples in the subspace is large while the intraclass distance is small (Figure 3(b)). Therefore, the proposed biomarkers derived from morphological features exhibited superiority over their original form; that is, our proposed biomarker extraction method was effective. Moreover, from Figures 3(c) and 3(d), we can see that the differences between pMCI and sMCI along the two dimensions in subspace were significant.

## 4. Discussion

In this work, we presented a novel biomarker extraction method based on subspace learning for the prediction of MCI-to-AD conversion. The developed biomarkers outperformed the competing biomarkers on the discrimination between pMCI and sMCI subjects. Moreover, the improvement from the developed biomarkers was not limited to a particular classifier but worked equally well for three different classifiers. In a word, this work provided a promising biomarker for the early diagnosis of AD.

### 4.1. Effectiveness Analysis of the Proposed Method

The good performance of our proposed method can be attributed to three reasons: (1) Effective subspace learning. We have demonstrated that the MCI subjects in original morphological feature space were high-dimensional and severely overlapped with each other. Therefore, subspace learning methods mapped multivariate MRI data of MCI subjects into a common subspace with fewer dimensions, where they were much easier to be distinguished.  Figure 3 clearly exhibits the efficacy of the space transformation. (2) The information of AD and NC subjects was employed. Compared with MCI subjects, the distances between intraclass samples are small while interclass samples are large for AD and NC subjects. Thus, it is easier to keep the neighborhood relationship between intraclass samples in subspace learning using AD and NC data. In addition, the utilization of AD and NC subjects instead of MCI subjects during subspace learning can avoid the double-dipping problem [56] in the classification of sMCI and pMCI. Therefore, it is reasonable to learn projection matrices using AD and NC data for MCI data, which was verified by the results in Table 4. (3) The self-weighted operation and weighted fusion were conducted. According to the projection matrices learned from AD and NC data, we mapped the thickness and volume data of MCI subjects into a common subspace. The feature representations of MCI subjects in the subspace, i.e., Fea_CT_ and Fea_CV_, were obtained. After that, we conducted the self-weighted operation on Fea_CT_ and Fea_CV_, to further amplify the differences between pMCI and sMCI. Although the cortical thickness and volume provided complementary information for the discrimination between pMCI and sMCI, the effect of them on classification is imbalanced; the more discriminative the morphological features are, the larger weights they should possess. Thus, we performed weighted fusion on thickness and volume-based features to obtain the final biomarkers. The results in Section 3 implied the effectiveness of our extracted biomarkers.

### 4.2. Influence of the Number of Auxiliary Data on Classification Accuracy

**Figure 4:**
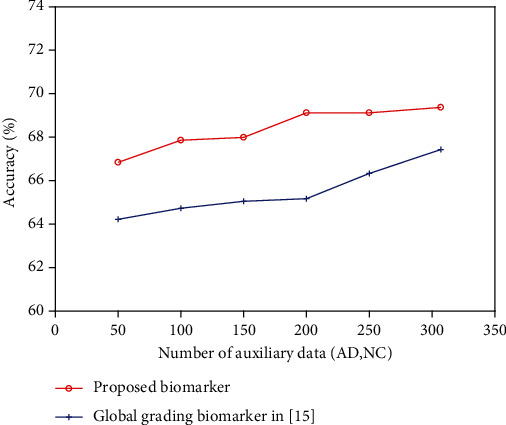
Classification accuracy of using different numbers of auxiliary data.

To study the influence of the number of auxiliary data on classification accuracy, we firstly used different numbers of auxiliary data to calculate the grading biomarker in [15] and the proposed biomarker, respectively, and then compared the differences of performance between them using SVM classifier with linear kernel. The number of auxiliary data varied from 50 to 250 with an increment of 50. For each specific number, we resampled the AD and NC subjects with the proportion of 1 : 1 for 10 times and calculated the average classification accuracy to avoid the sampling bias. The same procedure of 10-fold cross-validation and parameter optimization as Section 3 were conducted in the classification. The classification accuracies of two competing biomarkers with respect to different numbers of auxiliary data are illustrated in Figure 4. For comparison, we also plotted the classification accuracies of biomarkers computed by all auxiliary data. As shown in Figure 4, the classification performance of both two methods improves gradually with the increase of the number of auxiliary data, which verify the number of auxiliary data has an impact on classification performance of the biomarkers. In addition, the proposed biomarker outperforms the grading biomarker in [15] with different numbers of auxiliary data, which confirms the effectiveness of our proposed method.

### 4.3. Limitations

There are several limitations that should be addressed in the future work. Firstly, in our work, the CCA was adopted to maintain the correlation between the thickness features and volume features of the same image. And the graph regularization term was used to preserve the neighborhood relationship of samples in the subspace. However, other subspace learning methods, such as ICA, LDA, and LLE, should be further explored and validated in the biomarker extraction. Secondly, to map the MCI subjects into subspace, we learned the projection matrices only using the information of AD and NC subjects. It remains to be explored whether the performance can be improved by integrating the information of AD, NC, and MCI subjects during the projection matrices learning process. Thirdly, the proposed method took advantage of the limited morphological features, i.e., thickness and volume. As a matter of fact, different morphological features could reflect abnormal alterations of the brain from different perspectives, so they might provide complementary information for the early recognition of disease. More morphologies such as surface area [57], gyrus height [58], and local gyrification index [59] could be adopted to improve the classification performance.

## 5. Conclusion

In this paper, we developed the novel biomarkers based on subspace learning and the information integration of AD and NC subjects, which found a good feature representation from high-dimensional MRI data for predicting conversion from MCI to AD. The extracted biomarkers exhibited promising performance on discrimination between pMCI and sMCI, which validated the effectiveness of our proposed method. In addition, experimental results showed that the subspace learning was effective approach for finding satisfactory biomarkers and the information integration of AD and NC subjects was beneficial for the prediction of MCI-to-AD conversion.

## Figures and Tables

**Algorithm 1:**
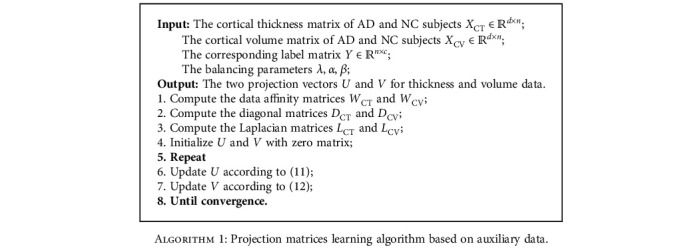
Projection matrices learning algorithm based on auxiliary data.

**Table 1:** Characteristics of the subjects.

Variables	NC	MCI		AD
		sMCI	pMCI	
No. of subjects (male/female)	165 (78/87)	95 (63/32)	126 (73/53)	142 (72/70)
Age	76.40 ± 5.37	74.94 ± 7.32	73.40 ± 9.25	76.10 ± 7.51
CDR	0	0.5	0.5	0.5/1
MMSE	29.19 ± 0.96	27.69 ± 1.73	26.49 ± 1.70	23.20 ± 2.01

CDR: Clinical Dementia Rating; MMSE: Mini-Mental State Examination.

**Table 2:** Classification results of two competing biomarkers using different classifiers.

Feature	Classifier	ACC (%)	*p* value	SEN (%)	SPE (%)	AUC
Global grading biomarker in [15]	Decision tree classifier	64.72	0.0082	69.30	58.56	0.6097
Proposed biomarkers		66.35		70.97	60.33	0.6216
Global grading biomarker in [15]	SVM classifier with RBF kernel	68.22	0.5169	83.35	48.03	0.6790
Proposed biomarkers		68.37		83.57	48.06	0.6800
Global grading biomarker in [15]	SVM classifier with linear kernel	67.43	<0.0001	70.04	63.96	0.6981
Proposed biomarkers		69.37		75.39	61.23	0.6951

**Table 3:** Comparison of classification results of all competing methods.

Feature	Classifier	ACC (%)	SEN (%)	SPE (%)	AUC
Original morphological features	SVM classifier with RBF kernel	62.92	71.23	51.72	0.6508
MFN in [13]	SVM classifier with RBF kernel	65.61	70.63	58.95	0.6670
Global grading biomarker in [15]	SVM classifier with linear kernel	67.43	70.04	63.96	0.6981
Proposed biomarker	SVM classifier with linear kernel	69.37	75.39	61.23	0.6951

**Table 4:** Classification performance of learning projection matrices using different data.

Data of learning projection matrices	Classifier	ACC (%)	*p* value	SEN (%)	SPE (%)	AUC
pMCI and sMCI data	Decision tree classifier	60.89	<0.0001	67.22	52.27	0.5318
AD and NC data		66.35		70.97	60.33	0.6216
pMCI and sMCI data	SVM classifier with RBF kernel	65.17	<0.0001	73.95	53.46	0.6495
AD and NC data		68.37		83.57	48.06	0.6800
pMCI and sMCI data	SVM classifier with linear kernel	64.78	<0.0001	67.59	60.91	0.6628
AD and NC data		69.37		75.39	61.23	0.6951

## Data Availability

The data comes from the Alzheimer's Disease Neuroimaging Initiative (ADNI) database (http://adni.loni.usc.edu/).
